# Glutathione Disulfide as a Reducing, Capping, and Mass-Separating Agent for the Synthesis and Enrichment of Gold Nanoclusters

**DOI:** 10.3390/nano11092258

**Published:** 2021-08-31

**Authors:** Qianqian Zhang, Junhua Wang, Zhao Meng, Rui Ling, Hang Ren, Weidong Qin, Zhenglong Wu, Na Shao

**Affiliations:** 1 College of Chemistry, Beijing Normal University, No. 19, XinJieKouWai Street, Beijing 100875, China; zhangqianqian121@126.com (Q.Z.); wjh15535439386@163.com (J.W.); 201921150027@mail.bnu.edu.cn (Z.M.); lggxdyjmm@163.com (R.L.); 13591706225@163.com (H.R.); 2 Analytical and Testing Center, Beijing Normal University, No. 19, XinJieKouWai Street, Beijing 100875, China

**Keywords:** glutathione disulfide, nanoclusters, enrichment, fluorescence, mass-separating agent

## Abstract

Water-soluble nanoclusters, which are facilely enrichable without changes in the original properties, are highly demanded in many disciplines. In this contribution, a new class of gold nanoclusters (AuNCs) was synthesized using glutathione disulfide (GSSG) as a reducing and capping agent under intermittent heating mode. The as-prepared GSSG–AuNCs had a higher quantum yield (4.1%) compared to the conventional glutathione-protected AuNCs (1.8%). Moreover, by simply introducing the GSSG–AuNC solution to acetonitrile at a volume ratio of 1:7, a new bottom phase was formed, in which GSSG–AuNCs could be 400-fold enriched without changes in properties, with a percentage recovery higher than 99%. The enrichment approach did not need additional instruments and was potentially suitable for large-scale enrichment of nanoclusters. Further, density functional theory calculations indicated that the hydrogen bonding between GSSG and acetonitrile plays a key role for the bottom phase formation. Our work suggests that the highly emissive GSSG–AuNCs possess great potential not only in fluorescent measurements but also in other scenarios in which high-concentration AuNCs may be needed, such as catalysis, drug delivery, and electronic and optical industries.

## 1. Introduction

Gold nanoclusters (AuNCs) are a family of molecular-like photoluminescent nanostructures typically composed of a few to approximately 100 Au atoms, which are less than 2 nm in size [1,2]. Unlike other nanomaterials that are synthesized with relatively strong reductants [3,4], water-soluble gold nanoclusters (AuNCs) are synthesized using weak reducing reagents, including phosphoniums, folic acid, amino acids, peptides, Good’s buffers, proteins, exosomes, and thiolates [5,6,7,8,9,10]. Among them, glutathione-protected AuNCs (GSH–AuNCs) have attracted extensive research interest. Compared with the conventional fluorescent dyes, fluorescent proteins, quantum dots, carbon nanodots, and upconverting lanthanide-doped nanoparticles, GSH–AuNCs have the advantages of low toxicity, excellent cell membrane permeability, and therefore good biocompatibility. Nonetheless, they possess a low quantum yield (QY) [11] and a low molar extinction coefficient that result in poor luminescence. For sensitive detection, additional strategies are often employed, such as lanthanide ion-induced enhancement [12], two-photon excited photoluminescence [13], and even enrichment of the nanoclusters [14]. In fact, enrichment is an indispensable step in the applications where high-concentration nanoclusters are needed, e.g., catalysis [15,16,17], therapy [18,19], and electronic and optical industries [20,21,22,23,24]. A number of concentration methods can potentially be used for this purpose, for instance, ultracentrifugation, ultrafiltration, reduced-pressure evaporation, chromatography, centrifugation, nanofluidic system, organic solvent-induced precipitation, and liquid–liquid extraction [25,26,27,28,29]. Of these, ultracentrifugation, ultrafiltration, and reduced-pressure evaporation are time consuming and laborious; chromatography, centrifugation, and nanofluidic systems are often restricted to small sample volumes; the nanoclusters obtained using organic solvent-induced precipitation are usually not readily redispersible; and the conventional liquid–liquid extraction, which often relays on ligand modification of the nanomaterials, changes the physico-chemical properties of the nanomaterials.

Glutathione disulfide (GSSG) is derived from the oxidation of two molecules of GSH; it contains a disulfide bond. Disulfide bonds are reductive according to the literature [30]. In this context, GSSG is a reductant, although it is weaker than GSH. However, unlike GSH, GSSG is seldom employed as a reductant in nanomaterial synthesis. Instead, it has been used as a capping agent for a number of nanostructures, such as Fe_3_O_4_ nanoparticles [31], gold nanorods [32], gold nanoclusters [33], and molybdenum disulfide [34].

It is well known that AuNC preparation requires weak reductants. In this context, it is meaningful to investigate the potential usefulness of GSSG as a reductant in AuNC synthesis. Herein, we report the one-pot, intermittent heating-mediated synthesis of high-luminescence AuNCs using GSSG as a reducing and capping agent. The QY of the obtained GSSG–AuNCs was significantly enhanced compared with that of conventional GSH–AuNCs. Further studies revealed the unique properties of the GSSG–AuNCs as compared with conventional GSH–AuNCs. Although the cores of GSSG–AuNCs and GSH–AuNCs are nearly identical, the GSSG–AuNC surfaces possess a higher content of Au–S motifs than what the GSH–AuNC surfaces do. Moreover, the GSSG–AuNC aggregates are packed in crystal, while the GSH–AuNCs show amorphism. Furthermore, the GSSG–AuNCs aggregate at a higher degree in aqueous solution.

Intriguingly, we found that by adding the as-prepared GSSG–AuNC aqueous solution into acetonitrile at an appropriate ratio, a new phase was formed, into which the AuNCs were nearly completely extracted, with an enrichment factor as high as 400-fold, whereas their chemical, photoluminescence (PL), and morphological properties were not changed. Further studies revealed that GSSG is a mass-separating agent responsible for the phase separation.

The highly emissive, biocompatible GSSG–AuNCs have attractive potential in fluorescent analysis and bioimaging, and the facile enrichability renders this kind of nanomaterial applicable in areas where high-concentration AuNCs are needed. Moreover, GSSG, a new mass-separating agent found by this work, may find its place not only in enrichment of nanoclusters but also in other disciplines such as separation and extraction.

## 2. Materials and Methods

### 2.1. Chemicals and Materials

Hydrogen tetrachloroaurate (III) tetrahydrate (HAuCl_4_·4H_2_O) was purchased from Sinopharm Chemical Reagents (Shanghai, China). Acetonitrile, chloroform, ethyl acetate, cyclohexane, tetrahydrofuran, methanol, sodium dihydrogen phosphate (NaH_2_PO_4_), and sodium hydroxide (NaOH) were obtained from Beijing Chemical Plant (Beijing, China). Glutathione (GSH), carbon tetrachloride, hexane, and glutathione disulfide (GSSG) were obtained from Aladdin (Shanghai, China). Hydrogen peroxide (H_2_O_2_), hydrochloric acid (HCl), and nitric acid (HNO_3_) were the products of Tongguang Fine Chemicals (Beijing, China). Triple-distilled water was used for all experiments.

### 2.2. Synthesis of GSSG–AuNCs and GSH–AuNCs

The glassware used for synthesis was first soaked in aqua regia for 5 h, then ultrasonically rinsed with triple-distilled water 5 times, and dried in air before use.

#### 2.2.1. Heating Modes

Two heating modes were employed in synthesis, viz., continuous and intermittent heating. For a typical continuous heating process, the reactants were held at 80 °C by keeping the round-bottomed bottle in a water bath. In a typical intermittent heating experiment, the round-bottomed bottle was placed in the water bath at 80 °C and ambient temperature, alternately, each for 4 h. The times needed for the reaction solution in the round-bottomed bottle to reach the desired temperatures were measured, being 14.5 min in the heating process, allowing the temperature of the reaction solution to increase from 24.7 °C to 80.0 °C (average 3.81 °C/min), and 13.4 min in the cooling process, during which the temperature of the reaction solution decreased from 80 °C to 24.7 °C (average 4.13 °C/min). For the cooling process, the room-temperature cooling water in the water bath was refreshed every 3 min until the temperature of the reaction solution reached equilibrium.

#### 2.2.2. Synthesis of GSSG–AuNCs

Under vigorous stirring, freshly prepared aqueous solutions of 8 mL of 20 mM HAuCl_4_ and 6 mL of 100 mM glutathione disulfide were mixed with 66 mL of triple-distilled water in a round-bottomed flask at ambient temperature. Two minutes later, a 24 h heating process, either under continuous mode or under intermittent heating mode, was initiated by placing the bottle in the 80 °C water bath under gentle stirring. After reaction, the precipitates were removed by centrifugation at 2000 rpm for 10 min, and the supernatant was stored at 4 °C until use.

#### 2.2.3. Synthesis of GSH–AuNCs

For the continuous heating mode, the procedures in the literature [35] were followed. Briefly, the freshly prepared aqueous solutions of 8 mL of 20 mM HAuCl_4_ and 2.4 mL of 100 mM glutathione were mixed first. The mixture was then diluted with triple-distilled water to 80 mL at room temperature, followed by a 24 h continuous heating under gentle stirring. For the intermittent heating mode, the parameters same as those of GSSG–AuNCs were adopted. The orange-emitting GSH–AuNCs obtained were stored at 4 °C until use.

### 2.3. Characterization 

UV–VIS absorption spectra were recorded on a TU-1901 UV–VIS spectrophotometer (Beijing Purkinje General Instrument, Beijing, China) using a 1 cm cell. PL spectra and phosphorescence decay experiments were performed on an FLS980 spectrometer (Edinburgh Instruments Ltd., Livingston, UK). The absolute QYs were measured with a C11347-11 Quantaurus-QY absolute PL quantum yield spectrometer (Hamamatsu, Japan). Transmission electron microscopy (TEM) images of the gold nanoclusters were taken on a Talos F200S transmission electron microscope (FEI, Hillsboro, OR, USA) operated at 200 kV. Dynamic light scattering (DLS) measurements were performed for colloidal solutions using a ZetaPlus laser light scattering system (Brookhaven, NY, USA). Matrix-assisted laser desorption/ionization–time-of-flight mass spectrometry (MALDI-TOF MS) was carried out on a MALDI-TOF/TOF 5800 analyzer (ABSciex, Framingham, MA, USA) equipped with a neodymium:yttrium–aluminum–garnet laser source emitting at a wavelength of 349 nm, using α-cyano-4-hydroxycinnamic acid (CHCA) as the matrix. X-ray photoelectron spectroscopy (XPS) was performed using an ESCSLAB 250XI X-ray photoelectron spectrometer (Thermo Fisher Scientific, Leicestershire, UK) equipped with a monochromatized Al-K𝛼 source at a power of 2721 eV. Inductively coupled plasma–atomic emission spectrometry (ICP-AES) measurements were carried out on a SPECTRO ARCOS EOP axial-view inductively coupled plasma spectrometer (SPECTRO Analytical Instruments GmbH, Kleve, Germany). The plasma power was 1.28 kW, and the coolant, auxiliary, and nebulizer flows were set at 13, 0.8, and 0.8 L/min, respectively.

### 2.4. Acetonitrile-Initiated Phase Separation

Typically, 1 mL of GSSG–AuNCs was transferred into a centrifuge tube containing 7 mL of acetonitrile. The mixture was shaken thoroughly for 10 s and then was allowed to stand for 2 h or was centrifuged at 2000 rpm for 10 min, followed by phase separation at ambient temperature. The upper phase was transferred to another clean centrifuge tube.

To investigate the luminescent properties of the AuNCs in the upper and lower phases after the enrichment, the acetonitrile in the solutions was removed by reduced-pressure evaporation at 70 °C. The residues were dissolved, separately, in 1 mL triple-distilled water and stored at 4 °C before analysis.

### 2.5. Density Functional Theory Calculation

Density functional theory (DFT) computations were performed following the protocols of a previous work using the Gaussian 16 package [36] with modifications. The geometric structures of GSSG–acetonitrile and GSH–acetonitrile complexes were optimized using the basis set of 6–31G(d,p) in water, and the basis set def2tzvp was employed to calculate the adsorption energy. The M06-2X method was adopted for all calculations. Based on the theory [37], the adsorption energy E_ads_ was evaluated by the following formula

(1) $$E_{ads}=E_{\left(ACN+GSSG/GSH\right)}-E_{ACN}-E_{\left(GSSG/GSH\right)}$$

where *E_(ACN+GSSG/GSH)_*, *E_ACN_*, and *E_(GSSG/GSH)_* represent the energies of the optimized combined structure (GSSG–ACN or GSH–ACN), acetonitrile, and GSSG or GSH, respectively.

## 3. Results and Discussion

### 3.1. Synthesis of GSSG–AuNCs

**Figure 1:**
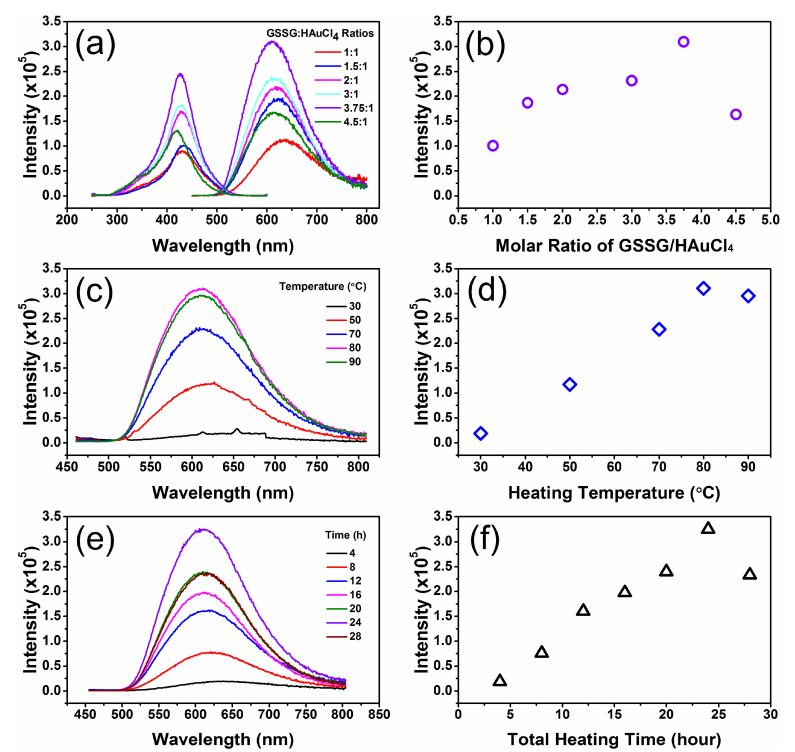
Optimization of the synthesis parameters. (**a**) Influence of the initial GSSG/HAuCl_4_ molar ratio on the excitation (left) and emission (right) of the GSSG–AuNCs; (**b**) variation of the emission maximum as a function of the GSSG/HAuCl4 molar ratio; (**c**) influence of the heating temperature on the emission spectra of the nanoclusters; (**d**) plot of emission intensities under different reaction temperatures; (**e**) effect of total heating time; and (**f**) dependence of emission intensity on the total reaction time. In the above experiments, the concentration of HAuCl_4_ was kept at 2 mM.

**Figure 2:**
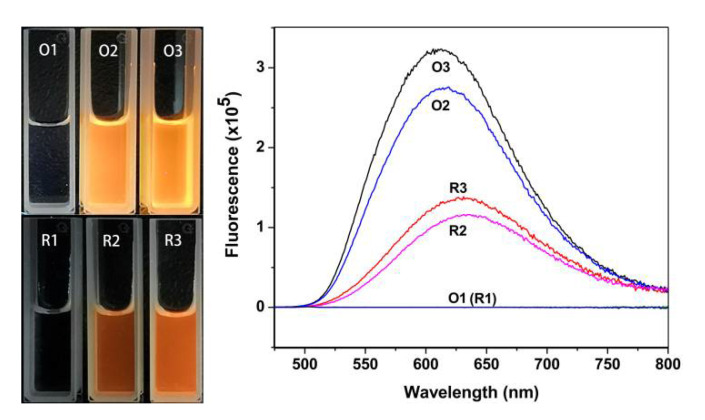
Photograph (**left panel**) and fluorescence emission spectra (**right panel**) of the AuNCs synthesized using GSH (denoted R) and GSSG (denoted O) as reducing and capping agents. Conditions: 1, reacting for 2 min at room temperature; 2, condition 1 first, then continuous heating at 80 °C for 24 h; 3, condition 1 first, then intermittent heating at 80 °C and at ambient temperature, alternately, each for 4 h, for a total time of 24 h. The photograph was taken under UV light at 365 nm. The synthesis conditions for R2 were adopted from the literature [35].

Experimental conditions such as the initial concentration ratio of the starting materials, heating temperature, heating time, and heating mode affected the luminescence properties of GSSG–AuNCs. Note that in the widely adopted protocols for preparing the GSH-protected AuNCs, the initial concentration of HAuCl_4_ was 2 mM [35]; the influence of the concentration ratio between GSSG and HAuCl_4_ in this experiment was investigated by keeping HAuCl_4_ at 2 mM, while varying the concentration of GSSG for rational comparison. Upon increasing the GSSG concentration, the emission intensity of the resultant GSSG–AuNCs was enhanced due to the increasing amount of luminescent nanoclusters generated, reaching a maximum at a GSSG:HAuCl_4_ ratio of 3.75:1 (Figure 1a,b). Further increasing the GSSG content resulted in reduced fluorescence of the solution because a large amount of precipitations were found at the bottom of the flask. High reaction temperature favors the formation of nanoclusters; the emission intensity, after reaction for 24 h, increased from 1.66 × 10^4^ at 30 °C to 2.31 × 10^5^ at 70 °C, reaching a maximum at 80 °C (Figure 1c,d). Regarding the heating time, enhanced luminescence was obtained with elongated reaction time until 24 h; further increasing the reaction time reduced the emission intensity (Figure 1e,f). It was observed that intermittent heating yielded stronger fluorescent AuNCs. For the GSSG–AuNCs obtained under continuous heating mode (trace O2, Figure 2), the emission intensity was 2.73 × 10^5^; however, the emission intensity was 3.23 × 10^5^ for the GSSG–AuNCs synthesized under intermittent heating mode (trace O3).

Disulfide bonds are reductive, and the literature suggests that in the presence of chloroauric acid (HAuCl_4_), a disulfide compound (denoted RSSR, for example) can be oxidized to RSSO_2_R, RSO_2_H, and RSO_3_H [30]. Similar to the GSH-mediated AuNC synthesis process, the reaction between HAuCl_4_ and GSSG involves two critical steps: (i) The chloroauric acid was mixed with excess GSSG at room temperature, and Au(III) was quickly reduced to Au(I) within 2 min (Au(I) was detected by XPS analysis; Figure S1a), forming Au(I)–GSSG complexes. (ii) The solution was then heated in a water bath, and Au(I)–GSSG was further reduced by GSSG, forming Au nanoclusters (denoted as GSSG–AuNCs; Au(0) was detectable by XPS; refer to Figure S1a), with the Au crystal cores being capped by Au(I)–GSSG motifs. The results obtained by altering the reaction temperature, reaction time, and heating mode reveal that the properties of AuNCs were influenced by the reaction kinetics. Two reactions took place in opposite ways, affecting the nanocluster structures during formation of the AuNCs. One is the reduction of the Au(I) ions to Au(0) atoms, and the other is the etching of the nanocluster cores by the thiolates [38]. After the nanoclusters were formed, excessive heating, whether by elevating the heating temperature or by prolonging the heating time, favors the etching reactions.

Based on the experiments, the strongest photoluminescent GSSG–AuNCs could be obtained through intermittently heating the mixture of 2 mM HAuCl_4_ and 7.5 mM GSSG for 24 h at 80 °C and ambient temperature alternately, each for 4 h. Unless otherwise stated, the GSSG–AuNCs obtained under optimal conditions were used throughout the experiments.

### 3.2. Photophysical Properties

Under a UV lamp, the Au(I)–GSH and Au(I)–GSSG complexes formed in the first step did not emit noticeable luminescence (Figure 2, left panel), whereas the GSH- and GSSG-protected AuNCs emitted red and amber fluorescence, respectively. The GSSG–AuNCs possessed maximal excitation and emission wavelengths at 424 nm and 610 nm, respectively. The photoluminescence lifetime measurement of the GSSG–AuNCs showed a microsecond-scale average lifetime (τ1 = 2.28 μs, 46.19%, τ2 = 9.45 μs, 53.81%, Figure S2), indicating that the luminescence is attributed to ligand-to-metal charge transfer (LMCT) or ligand-to-metal–metal charge transfer (LMMCT) that generates radiative relaxation via a metal-centered triplet state [39]. However, the luminescence intensities of the GSH-protected AuNCs obtained under both continuous and intermittent heating modes were weaker (Figure 2, traces R2 and R3). The absolute QY for GSH–AuNCs obtained under optimal conditions was determined to be 1.8%, which was similar to the reported values, i.e., 1% [40], 2% [41,42], and 1.2% [11]. In contrast, the GSSG–AuNCs possessed significantly higher luminescent efficacy, offering an absolute QY of 4.1%.

### 3.3. Morphological and Compositional Investigations

To understand the mechanism of higher QYs of the GSSG–AuNCs, TEM, DLS, MALDI-TOF MS, and XPS experiments were carried out, and the results were carefully analyzed.

**Figure 3:**
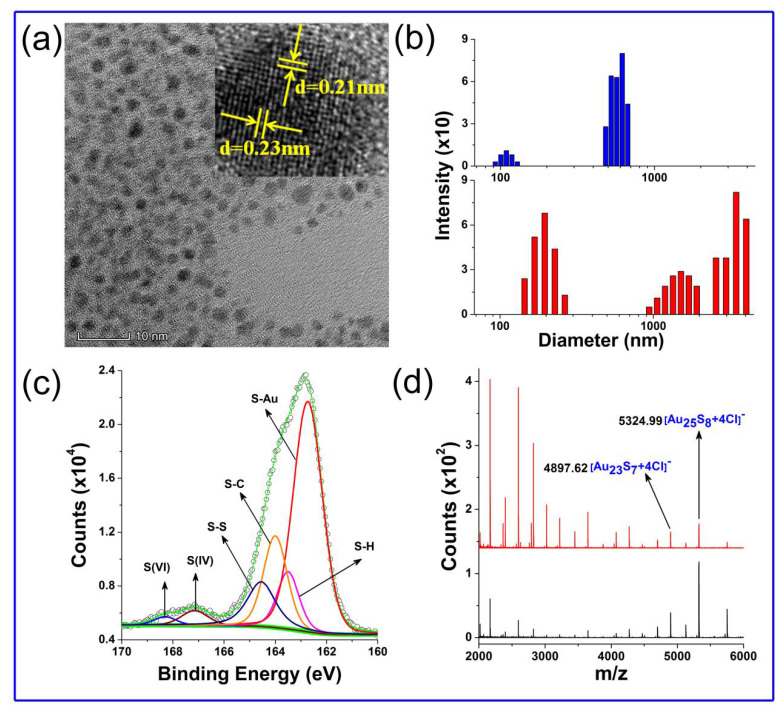
(**a**) HR-TEM image of the GSSG–AuNCs (inset shows the lattice fringe of the nanoclusters). (**b**) DLS spectra of GSH–AuNCs (upper) and GSSG–AuNCs (bottom). (**c**) XPS spectrum of S 2p of GSSG–AuNCs. Peak identities: 168.2 eV, oxidized sulfur, S (VI); 167.1 eV, oxidized sulfur, S (IV); 164.5 eV, disulfide bond, S–S; 164.0 eV, sulfur–carbon bond, S–C; 163.5 eV, sulfur–hydrogen bond, S–H; 162.8 eV, sulfur–gold bond, S–Au. (**d**) MALDI-TOF mass spectra of GSSG–AuNCs (upper) and GSH–AuNCs (bottom).

The TEM image (Figure 3a) showed that the as-prepared GSSG–AuNCs were spherical, with an average diameter of 1.97 ± 0.25 nm. As observed by high-resolution transmission electron microscopy (HR-TEM), the lattice fringes of the GSSG–AuNCs were well resolved (inset, Figure 3a). The interplanar distances of 0.23 nm and 0.21 nm can be assigned to the (111) and (200) lattice planes of gold nanocrystals, respectively. However, although the GSH–AuNCs possess a similar size and morphology as GSSG–AuNCs, their lattice stripes are hard to recognize (Figure S3). The TEM results partially interpret the slightly shorter emission wavelength of the GSSG–AuNCs (610 nm) relative to that of the GSH–AuNCs (630 nm, Figure 1). As proposed by other researchers [43], disruption of the crystal packing leads to red shift of the nanocluster photoluminescence.

DLS measurements showed that the hydrodynamic diameter of the GSH–AuNCs was approximately 600 nm, while hydrodynamic diameters larger than 1000 nm were detected in a significant portion of the GSSG–AuNCs (Figure 3b). Although it is difficult to determine the size distribution of the AuNCs from the diagram because the scattered light intensity of individual particle increases dramatically with the particle size [44], we can deduce from the results that the GSSG–AuNCs possess a higher aggregation degree in aqueous solution. The dense aggregation of GSSG–AuNCs increases intra- and inter-complex aurophilic Au(I)–Au(I) interactions, while restraining intramolecular vibrations and rotations of the Au(I)–GSSG complexes, and as a consequence, enhanced photoluminescence can be obtained [35].

The molecular compositions of AuNCs were determined by MALDI-TOF MS under negative ion mode. Note that the MALDI analysis of water-soluble nanoclusters is challenging; nevertheless, the spectra of the AuNCs were successfully recorded (Figure 3d). The most abundant peak at *m*/*z* 5324.99 can be assigned to [Au_25_S_8_ + 4Cl]^−^, and a series of peaks with *m*/*z* spacings of 32 and 197 are the consequences of the loss of [S] and [Au], respectively. The spectra indicate that GSSG–AuNCs and GSH–AuNCs have similar ionization and core fragmentation patterns, implying that they have the same core composition. However, the GSSG–AuNCs had relatively stronger fragment intensities in the *m*/*z* range of 2000–4000, while the GSH–AuNCs had a higher abundance for fragments beyond *m*/*z* 5000. The results indicate that the water-soluble AuNCs obtained are a mixture of nanoclusters with different molecular weights; this observation agrees with the previous reports on GSH–AuNCs [45]. Moreover, previous research based on GSH–AuNCs revealed that the length of the Au(I)–thiolate motif varies with the nanocluster molecule size and that the QY generally increases with the decreasing nanocluster molecular weight [39]. Our results are in line with the reports. The GSSG–AuNCs, which possess relatively high abundance of smaller fragments, offer higher QYs.

X-ray photoelectron spectroscopy (XPS) studies depicted two core peaks with S 2p doublets (Figure 3c). The peak of lower binding energy was deconvoluted into four peaks centered at 162.7, 163.5, 164.0, and 164.5 eV, which were ascribed to S–Au, S–H, S–C, and S–S bonds, respectively. Using a similar approach, two peaks at higher binding energies of 167.1 and 168.18 eV corresponding to S (IV) and S (VI) could be deduced. The best fit of the data revealed that the relative proportion of S–Au bonds in the GSSG–AuNCs was 2.58%, higher than that in the GSH–AuNCs (0.697%, Figure S4). This result partially contributes to the stronger PL of the GSSG–AuNCs, because it is well known that aggregation of the Au–S motifs is one of the main sources of the Au nanocluster luminescence.

We also recorded Au 4f XPS spectra in GSSG–AuNCs and GSH–AuNCs for comparison (Figure S1). The Au 4f XPS peaks in GSSG–AuNCs showed lower binding energies. For example, in the 4f_7/2_ spectra range, the binding energies of Au(0) and Au(I) were 84.3 and 84.7 eV, respectively, in the GSH–AuNCs; they shifted to 83.6 and 84.5 eV, respectively, in the GSSG–AuNCs. It is well known that Au(0) can interact with electron-donating thiol and disulfide groups. Our experiments suggest that the Au(0)–disulfide interaction is stronger. Similarly, the low energy shift of Au(I)-binding energy in GSSG–AuNCs reveals that the Au(I)–GSSG interaction is stronger than the Au(I)–GSH interaction. The stronger Au(0)–disulfide and Au(I)–GSSG interactions favored the high aggregation degree of the nanoclusters in the aqueous phase that was observed in the DLS measurements.

The unique properties of the synthesized GSSG–AuNCs, —i.e., regularly packed nanocluster crystals that are more heavily aggregated in water, higher distribution of smaller nanoclusters, and more abundant Au(I)–GSSG motifs that cap the nanocluster cores,—gave rise to the blue-shifted, intensity-enhanced fluorescent emission of GSSG–AuNCs.

### 3.4. Enrichment and Redispersion of GSSG–AuNCs

**Figure 4:**
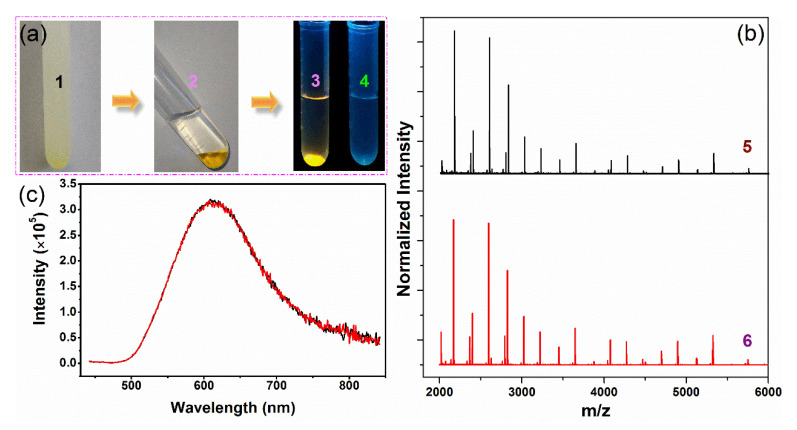
(**a**) Schematic illustration of the formation of the new phase and the enrichment of GSSG–AuNCs: (1) A turbid solution formed after mixing the GSSG–AuNC solution with acetonitrile; (2) a new phase formed at the bottom of the tube; (3) irradiation with 365 nm UV light; and (4) the upper acetonitrile-rich phase irradiated under the same conditions for comparison. (**b**) MALDI-TOF/TOF MS spectra of the GSSG-AuNCs before (5) and after (6) enrichment. (**c**) Photoluminescent spectra of the GSSG–AuNCs before (red line) and after (black line) enrichment.

Organic solvents are often used to purify and concentrate water-soluble AuNCs through precipitation. We found that when acetonitrile was mixed with the aqueous GSSG–AuNC solution at certain volume ratios (the optimum was 7:1), a new liquid phase, instead of precipitates, was formed at the bottom of the centrifuge tube (Figure 4). Approximately 2.5 μL of the new phase was obtained from 1 mL of the original aqueous GSSG–AuNC solution, offering an enrichment factor of 400-fold. The new phase had good fluidity. The composite viscosities of the bottom phase between 25 and 50 °C, measured by rheological performance tests, were about 20 times the viscosity of water (Figure S5).

The fluorescence emission intensities of the AuNCs collected from the upper acetonitrile-rich phase and the bottom phase differed dramatically (Figure S6), with count readings of 2.40 × 10^3^ and 2.22 × 10^5^, respectively, suggesting that the majority of the GSSG–AuNCs were extracted to the bottom phase. Moreover, experiments indicated that the GSSG–AuNCs recovered from different phases shared similar fluorescence spectra (Figure 4c), fragment patterns (Figure 4b), and morphologies (Figure S7), implying that the properties of the GSSG–AuNCs did not change significantly after phase separation and the simultaneous enrichment process.

The main compositions of the typical bottom phase and the corresponding upper phase, which were formed by mixing 10 mL of GSSG–AuNCs with 70 mL of acetonitrile, were analyzed by gas chromatography, capillary electrophoresis, and ICP-AES. The bottom phase contained 10.3% (*v/v*) acetonitrile, 20.2% (*v/v*) water (Figure S8 and the corresponding description), and 137.3 mM free GSSG (Figures S9 and S10 show the representative electropherograms), the corresponding values in the upper phase being 87.3%, 12.4%, and 7.6 mM, respectively. Moreover, according to AES, 99.62% ± 0.28% (*n* = 3) of the Au element was distributed in the bottom phase (Table S1 of Supporting Information), confirming the photoluminescence measurements.

The results suggest that our method can provide quick enrichment of nanomaterials with a high enrichment factor and recovery as compared with the reported methods (Table 1). Unlike enrichment approaches that are based on nanofluidic devices [14,46], chromatography [28], or high-speed centrifugation [29], our method needs no sophisticated instruments. Importantly, by simply mixing the aqueous AuNC solution and acetonitrile and allowing the mixture to stand for 2 h, phase separation can be accomplished. Therefore, the method can be facilely applied to large-scale enrichment of GSSG–AuNCs.

Moreover, the bottom phase was readily redispersed in triple-distilled water. Interestingly, the redispersed GSSG–AuNCs can be re-enriched using acetonitrile following the same procedures. According to the ICP-AES measurements, the overall recovery of Au element in the 100 μL lower phase through three redispersion/re-enrichment cycles was 92.85%.

### 3.5. GSSG as the Mass-Separating Agent

To gain a deep understanding of the mechanism of phase separation, 20 mL of the aqueous GSSG–AuNC suspension was dialyzed (membrane dialyzer, 2 kDa cut off, Solarbio, Beijing, China) against 450 mL (150 mL × 3 times) of triple-distilled water. The nanoclusters trapped in the dialysis bag and the matrices (dialysate) outside were collected separately, evaporated to dryness to remove acetonitrile, and then dissolved in 20 mL of triple-distilled water, —denoted as dialyzed nanocluster solution and dialyzed matrix solution, respectively. At a volume ratio of 1:7, the addition of the dialyzed nanocluster solution to acetonitrile resulted in precipitates; conversely, a new phase was formed upon mixing the dialyzed matrix solution with acetonitrile. The experimental results implied that the residual GSSG in the original GSSG–AuNC solution may play a crucial role in phase separation.

To verify the hypothesis, 10 mL of 7.5 mM GSSG solution was mixed thoroughly with 70 mL of acetonitrile. A colorless new phase was found at the bottom of the bottle, suggesting the feasibility of GSSG as a mass-separating agent. It is widely accepted that hydrogen bonding plays a key role in aqueous two-phase separation. In the GSSG–AuNC solution, residual GSSG and its oxidization products formed hydrogen bond networks with water molecules. GSSG is sparingly soluble in acetonitrile. However, acetonitrile, which is fully miscible with water at room temperature, can form hydrogen bonds with the GSSG species in the aqueous GSSG–AuNC solution. In the presence of largely excessive acetonitrile, acetonitrile molecules might replace the water molecules of the GSSG–water networks in the aqueous nanocluster solution. As a result, some water molecules were “pushed” to the upper acetonitrile-rich phase.

The potential application of GSSG as a mass-separating agent was investigated by enriching the GSH–AuNCs. To this end, 0.2 mL of 30 mM GSSG was mixed with 0.8 mL of GSH–AuNCs and was then introduced to 7 mL acetonitrile and shaken thoroughly, and a yellow droplet of ca. 5 μL was found at the bottom of the tube after centrifugation. The ICP-AES test indicated that 98.32% of the Au species were distributed in the lower phase.

### 3.6. Mechanism Studies

Since GSH is also sparingly soluble in acetonitrile, and can form hydrogen bonds with acetonitrile in aqueous solution, experiments were carried out to test whether adding GSH–AuNC solution to acetonitrile could form a new liquid phase. It was observed that mixing GSH–AuNC solutions of different pH values (4.0–11.0) with acetonitrile at various volume ratios (1:15–1:1) resulted in precipitates. Similar results were obtained upon adding GSH solutions to acetonitrile.

**Figure 5:**
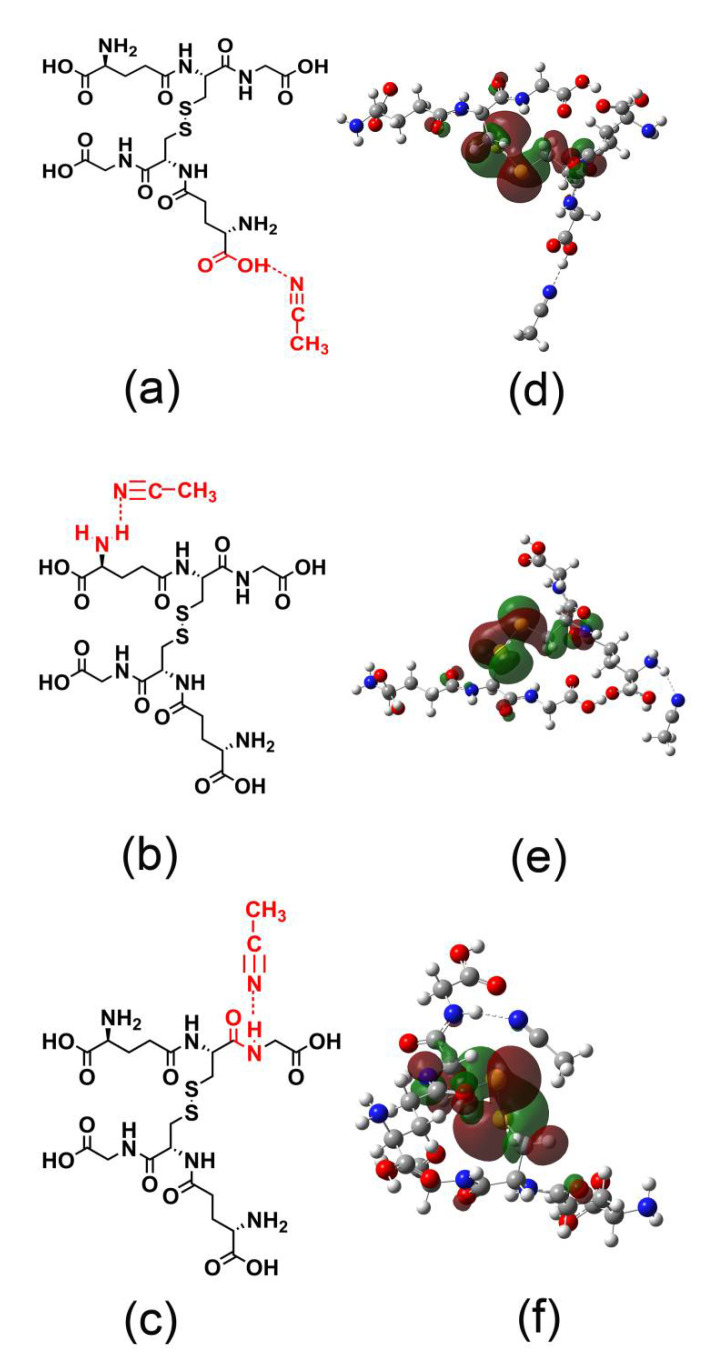
Hypothesized hydrogen bonds formed between acetonitrile and the GSSG functional groups: (**a**) carboxyl group, (**b**) amine group, and (**c**) amide group. (**d**–**f**) Corresponding frontier molecular orbitals of the charge transfers for (**a**–**c**).

GSSG differs from GSH chemically [48]. The more flexible molecular chain of GSSG affects the balance of the molecular conformational and intermolecular energy [49], which are key factors affecting the onset of nucleation and the subsequent precipitation. In addition, the pKa values of GSH are reported to be 2.12 (–CO_2_H), 3.59 (–CO_2_H), 8.75 (–SH), and 9.65 (–NH_3_^+^) [50], considerably different from the corresponding pKa values of GSSG, which are reported to be 1.80, 2.39, 3.29, and 3.92 for the four carboxyl groups and 8.94 and 9.68 for the protonated amine groups [51]. The differences affect the protonation/deprotonation of the amine and carboxyl groups and, consequently, the hydrogen-bonding capabilities, which greatly dictate whether the molecule can act as a mass-separating agent [52]. For example, GSSG in aqueous solution can form intramolecular hydrogen bonds upon the assistance of two water molecules, while GSH cannot [37]. Further, the chemical differences also influence their interaction with acetonitrile. Our DFT simulations indicate that the –COOH, –NH_2_, and –CONH groups of both GSSG and GSH molecules can form hydrogen bonds with acetonitrile in aqueous solution (Figure 5 and Figure S11). However, the adsorption energy of acetonitrile to the carboxyl is the strongest, which are calculated to be −6.15 and −5.99 kcal/mol for GSSG and GSH, respectively (Table 2), implying that the hydrogen bond between GSSG and acetonitrile is more stable. Our simulations and observations suggest that the stronger hydrogen bonding between GSSG and acetonitrile molecules stabilizes the bottom phase, preventing the formation of precipitates.

## 4. Conclusions

We synthesized new AuNCs using GSSG as both a reducing and a capping agent. Although the GSSG–AuNCs shared similar molecular compositions and morphologies with GSH–AuNCs, the former had a higher distribution of low-molecular-weight nanoclusters, possessed more surface Au–thiol ligands, were more regularly packed in the solid state, and were more heavily aggregated in aqueous solution. These characteristics rendered the GSSG–AuNCs significantly strongly photoluminescent with a QY considerably higher than that of GSH–AuNCs. Moreover, using GSSG as a mass-separating agent, the as-prepared GSSG–AuNCs in aqueous solution could be facilely enriched by as high as 400-fold, with recoveries higher than 99%, by simply mixing with acetonitrile at proper volume ratios. Furthermore, the enriched GSSG–AuNCs retained their original morphological and chemical properties and could be readily redispersed in water. The unique properties of the GSSG–AuNCs enable them to be potentially applied not only in fluorescent imaging but also in catalysis, drug delivery, and electronic and optical industries. Importantly, GSSG, a new mass-separating agent found by this work, may find its place not only in enrichment of nanoclusters but also in other disciplines such as separation and extraction.

## Figures and Tables

**Table 1:** Comparison of the performances of nanomaterial enrichment approaches.

Approach	Nanomaterials	Time	Enrichment Factor	Recovery	Ref.
Magnet solid-phase extraction	TiO_2_ nanoparticles	25 min	400	91.0–102.0%	[47]
Nanofluidic device under electroless actuation mode	Polystyrene nanobeads	60 min	200	n/a	[14]
Brownian ratchet-based nanofluidic system	100 nm polystyrene beads	17 min	27	n/a	[46]
C-18 column adsorption ligand-assisted liquid– liquid extraction	10 nm gold nanoparticles	n/a	250	68.4–99.4%	[28]
Ultracentrifugation	Curcumin–chitosan nanocomplexes	>20 min	n.a.	48%	[29]
Acetonitrile-initiated phase separation	Gold nanoclusters	10 min	400	99.62%	This method

**Table 2:** Adsorption energies of acetonitrile on GSSG and GSH in aqueous solution (kcal/mol).

	GSSG	GSH
–COOH	−6.15	−5.99
–NH_2_	−2.04	−3.89
–CONH	−4.46	−3.12

## Data Availability

All data generated and analyzed during this study are included in this paper and the attached supporting information.

## Supplementary Materials

The following are available online at https://www.mdpi.com/article/10.3390/nano11092258/s1, Table S1. Au (μg) content in the original GSSG–AuNC solution, upper phase and bottom phase; Figure S1. XPS spectra of Au 4f of GSSG–AuNCs (a) and GSH-AuNCs (b); Figure S2. Fluorescence lifetime decay profiles of GSH–AuNCs (a) and GSSG–AuNCs (b); Figure S3. TEM image of the GSH–AuNCs; Figure S4. XPS spectra of S 2p of GSH–AuNCs; Figure S5. Comparison of the composite viscosity between water (filled blue square, refer to the left Y axis) and the bottom phase (filled red circle, refer to the right Y axis); Figure S6. Photoluminescent spectra of the upper acetonitrile-rich phase (black line) and the bottom GSSG–AuNC-enriched phase (red line); Figure S7. TEM images of the GSSG–AuNCs before (a) and after (b) enrichment; Figure S8. Representative gas chromatogram of the bottom phase; Figure S9. Capillary electrophoresis of the standard mixture of GSH (1) and GSSG (2); Figure S10. Representative electropherograms of the upper acetonitrile-rich phase (a) and the bottom phase (b); Figure S11. Schematic drawings showing the hypothesized hydrogen bonds formed between acetonitrile and the GSH functional groups.
