# Supplementary material for: Glutathione Disulfide as a Reducing, Capping, and Mass-Separating Agent for the Synthesis and Enrichment of Gold Nanoclusters
Source: Nanomaterials (Basel). 2021 Aug 31;11(9):2258. doi: 10.3390/nano11092258 (PMC8472339; doi:10.3390/nano11092258)
Supplement: Supplementary file 1 [file nanomaterials-11-02258-s001.zip › nanomaterials-1359619-supplementary.pdf]

Supporting Information

Article

Glutathione Disulfide as a Reducing, Capping, and Mass-Separating Agent for the Synthesis and Enrichment of Gold Nanoclusters

Qianqian Zhang [1], Junhua Wang [1], Zhao Meng [1], Rui Ling [1], Hang Ren [1], Weidong Qin [1,*], Zhenglong Wu [2,*] and Na Shao [1,*]

[1] College of Chemistry, Beijing Normal University, No. 19, XinJieKouWai Street, Beijing 100875, China; zhangqianqian121@126.com (Q.Z.); wjh15535439386@163.com (J.W.); 201921150027@mail.bnu.edu.cn (Z.M.); lggxdyjmm@163.com (R.L.); 13591706225@163.com (H.R.)

[2] Analytical and Testing Center, Beijing Normal University, No. 19, XinJieKouWai Street, Beijing 100875, China

* Correspondence: qinwd@bnu.edu.cn (W.Q.); wuzl@bnu.edu.cn (Z.W.); shaona@bnu.edu.cn (N.S.)

ICP-AES analysis of the Au content in the upper- and bottom phases

Ten milliliters of the AuNCs solution were introduced to 21 mL of acetonitrile. After phase separation, the upper phase was evaporated under reduced pressure until ca. 1 mL was left. The bottom phase was topped up with triple distilled water to 2 mL. Then, the pretreated upper- and bottom phases were transferred separately to two 30-mL precleaned Teflon digestion vessels (Xi'an Gauge Instrument, Shaanxi, China). To each vessel, 2 mL of nitric acid and 2 mL of hydrogen peroxide were added. The vessels were put into an oven that was preheated to 180 °C for digestion. After the reaction, each product was diluted with triple distilled water to a volume of 50 mL prior to analysis. The experiment was repeated in triplicate.

Table S1 Au (μg) content in the original GSSG-AuNC solution, upper phase and bottom phase

	Original solution	Upper phase	Bottom phase	Recovery [a)]
1	2340.00	5.52	2338.00	99.91%
2	2358.00	5.95	2340.00	99.24%
3	2346.00	5.34	2339.00	99.70%
Average				99.62%±0.28%

[a)] Recoveries were calculated based on the Au content found in the bottom phase and the Au content detected in the original solution.

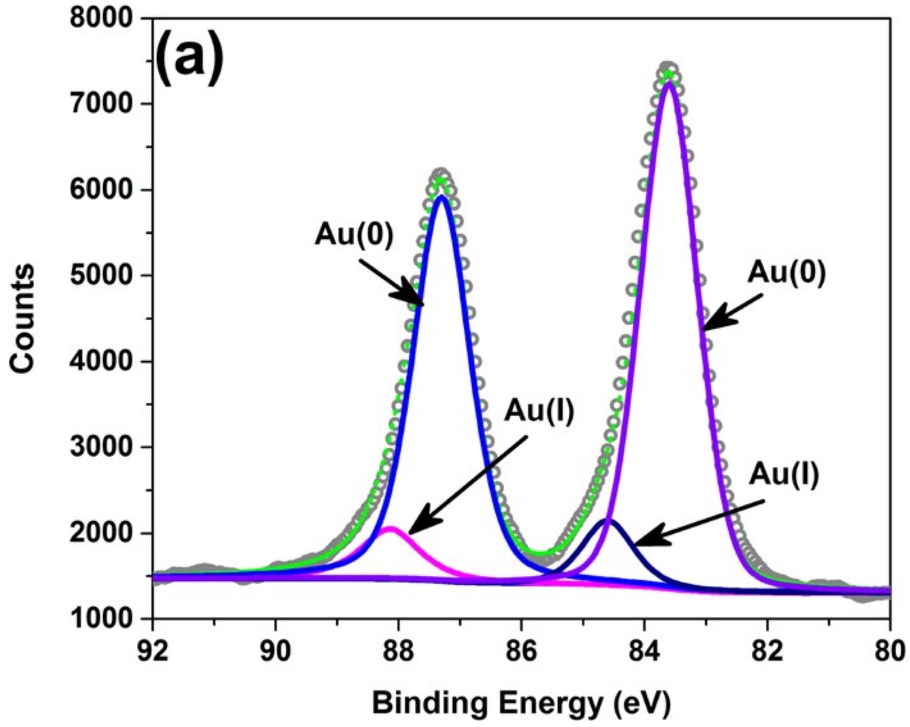

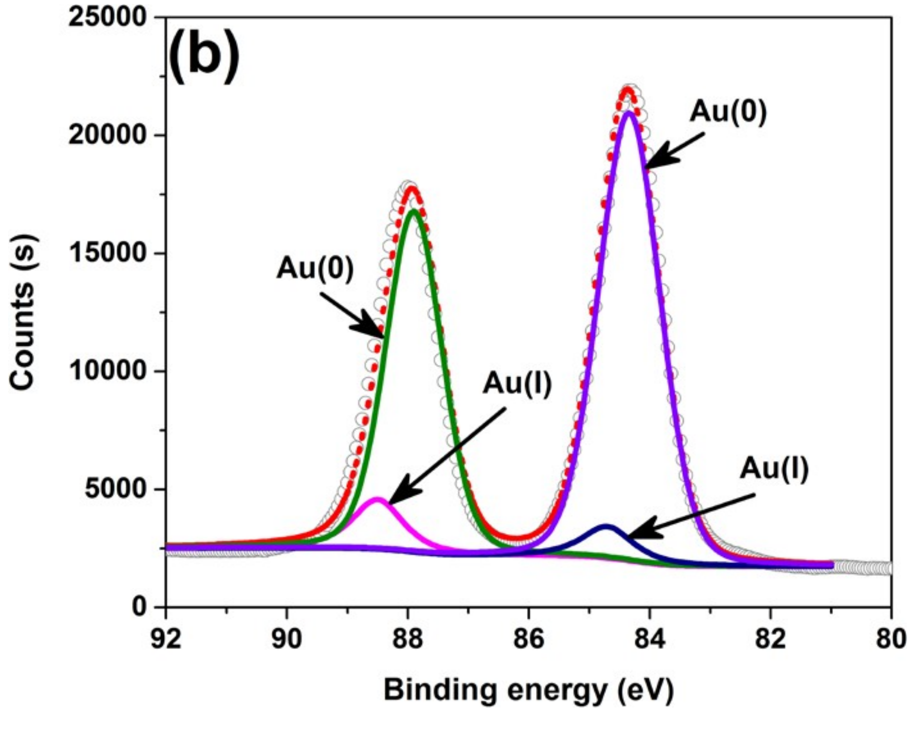

Figure S1 XPS spectra of Au 4f of GSSG-AuNCs (a) and GSH-AuNCs (b). Binding energies (eV) in GSSG-AuNCs: Au $4f_{7/2}$: Au(0), 83.6; Au(I), 84.5; Au $4f_{5/2}$: Au(0), 87.3; Au(I), 88.1. Binding energies (eV) in GSH-AuNCs: Au $4f_{7/2}$: Au(0), 84.3; Au(I), 84.7; Au $4f_{5/2}$: Au(0), 87.9; Au(I), 88.5.

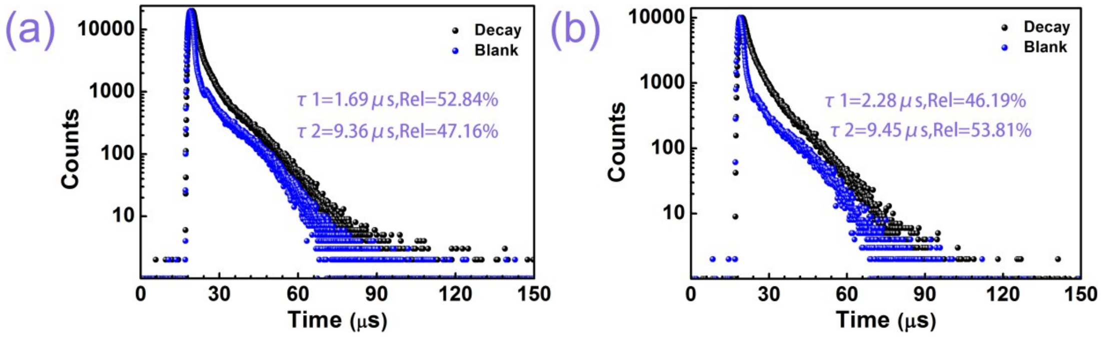

Figure S2 Fluorescence lifetime decay profiles of the GSH-AuNCs (a) and GSSG-AuNCs (b).

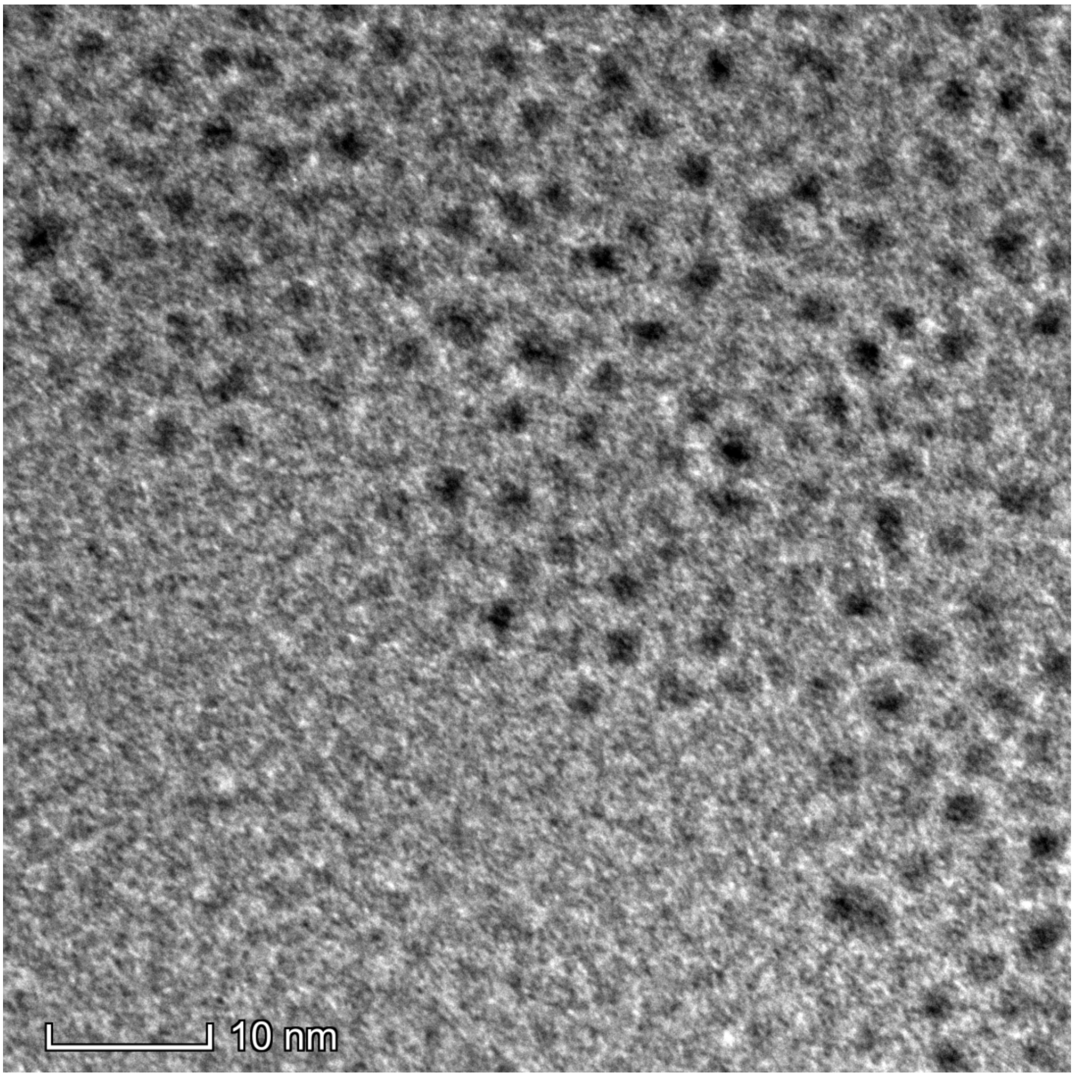

Figure S3 TEM image of the GSH-AuNCs.

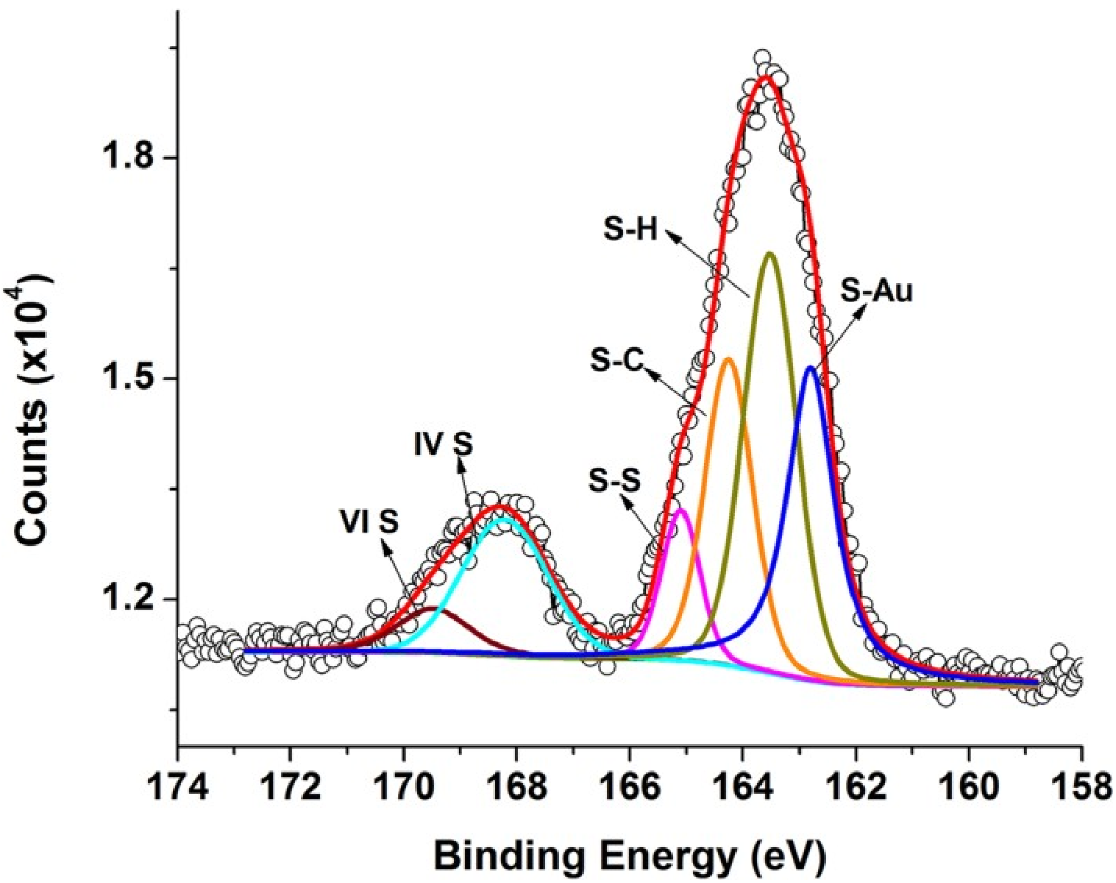

Figure S4 XPS spectra of S 2p of GSH-AuNCs.

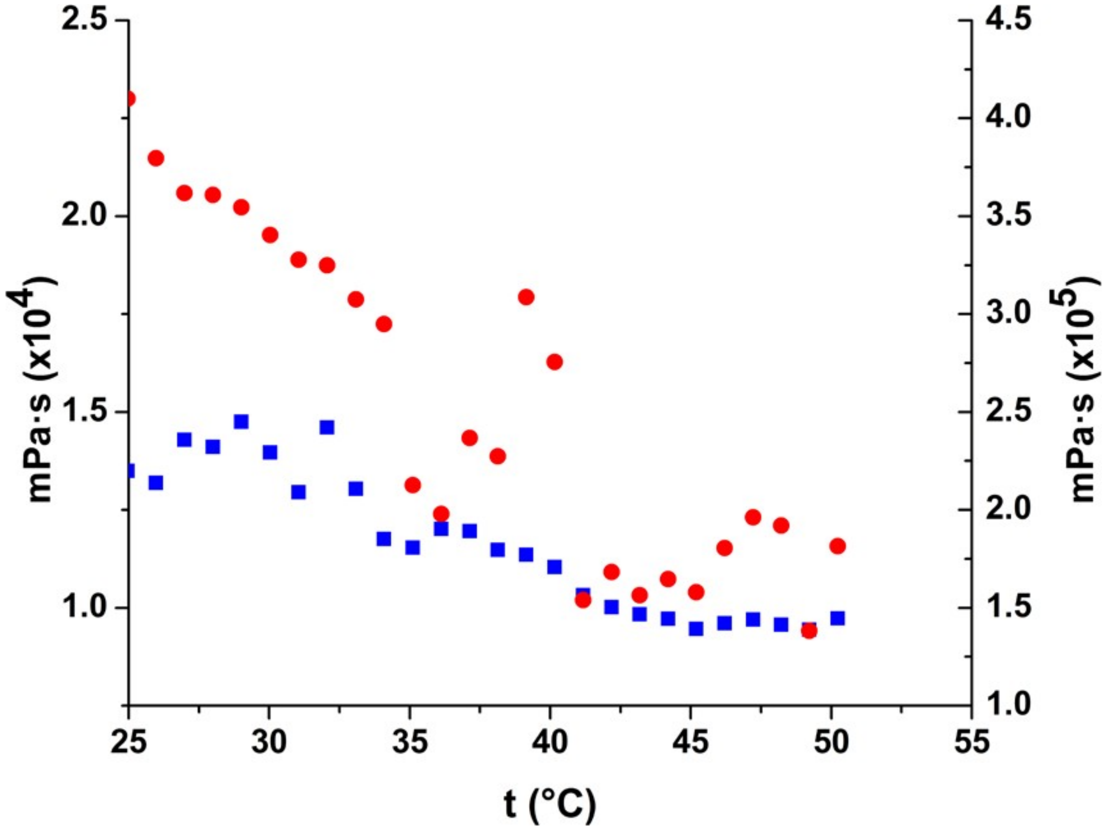

Figure S5 Comparison of the composite viscosity between water (filled blue square, refer to the left Y axis) and the bottom phase (filled red circle, refer to the right Y axis).

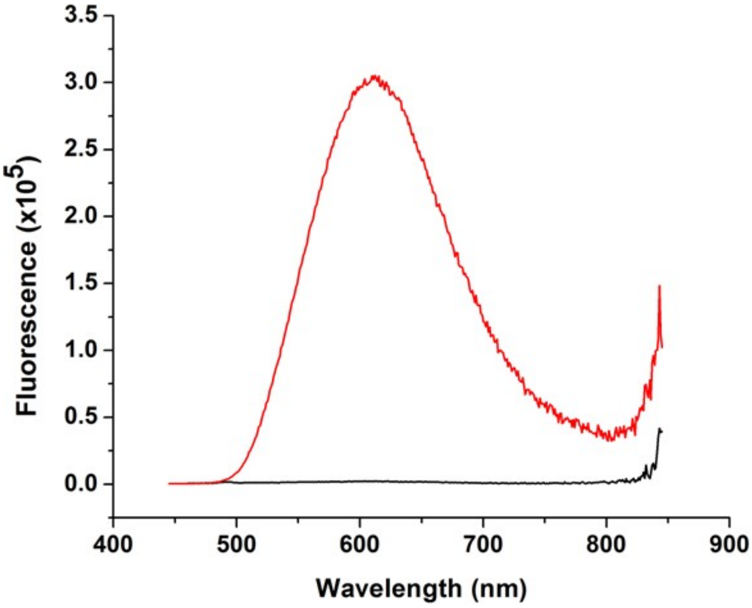

Figure S6 Photoluminescent spectra of the upper acetonitrile-rich phase (black line) and the bottom GSSG-AuNC-enriched phase (red line). Excitation wavelength: 424 nm. The fluorescence of the upper- and bottom phases was studied by first removing the acetonitrile through reduced-pressure evaporation and then adding triple distilled water to a volume equal to that of the original aqueous GSSG-AuNC solution used for phase separation.

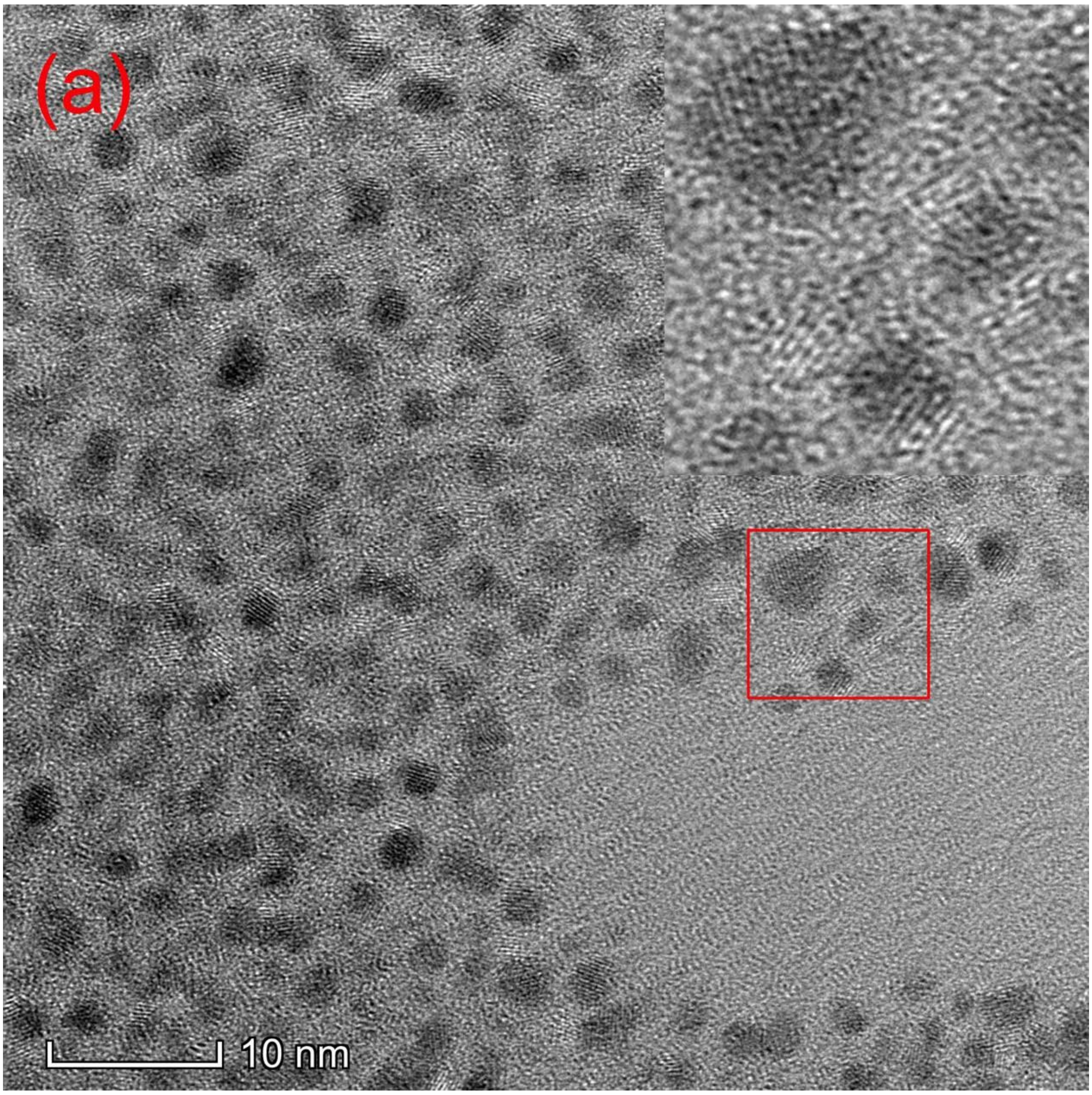

(a)
10 nm

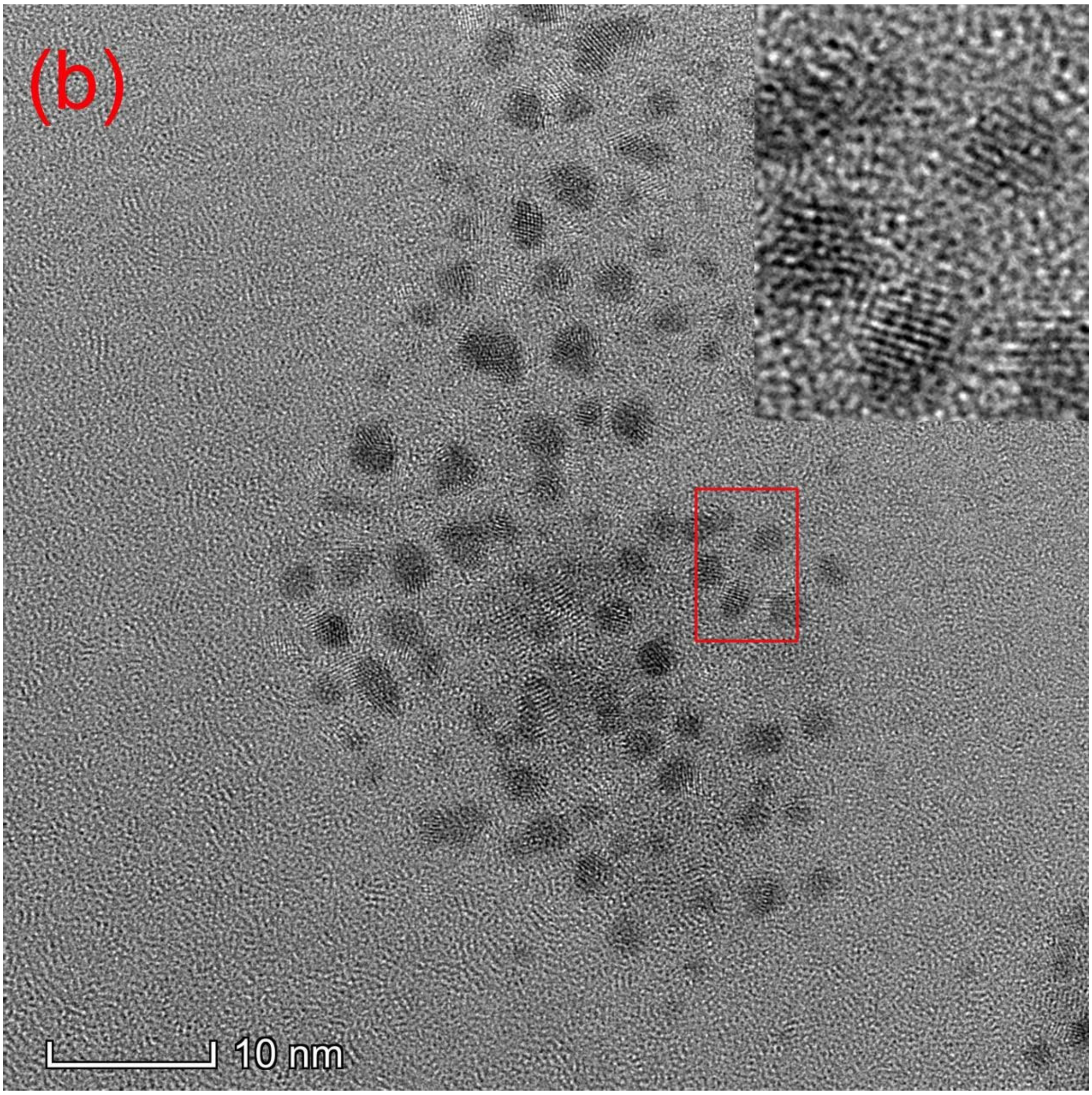

Figure S7 TEM images of the GSSG-AuNCs before (a) and after enrichment (b).

Gas chromatography

The acetonitrile and water contents in the bottom phase were determined by gas chromatography-thermal conductivity detector (GC-TCD, Varian, Walton-on Thames, UK). The bottom phase was diluted to 1:9 (V/V) with n-butanol. Using hydrogen as the carrier gas in constant flow rate mode on a polar packed column (GDX-103), chromatographic separation was performed at a flow rate of 30 mL/min. The oven temperature was maintained at 140 °C, the inlet and detector temperatures were set to 180 °C, the inlet pressure was 35.8 KPa, the bridge current was 100 mA, and the injection volume was 1 μL.

The calibration curves for water and acetonitrile were established on a series of standard mixtures of varying concentrations, and the volume fractions of acetonitrile and water in the bottom phase were determined to be 10.3% and 20.2%, respectively.

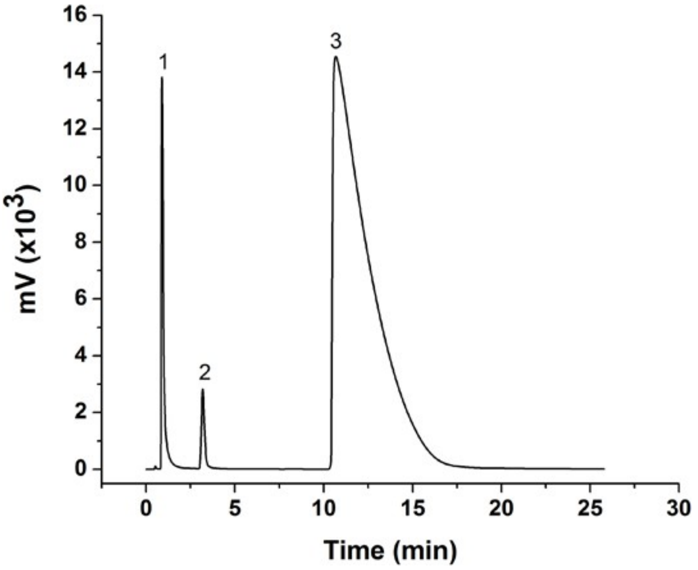

Figure S8 Representative gas chromatogram of the bottom phase. Peak identities: 1, water; 2, acetonitrile; 3, n-butanol.

Capillary electrophoresis

Capillary electrophoresis experiments were conducted on a CE-UV system composed of an HB-P303-1AC high-voltage power supply (Hengbao, Tianjin, China) and a CE-10 UV detector (Johnson Separation Science, Liaoning, China), which was operated at 365 nm. The signal from the detector was collected by a desktop computer through a two-channel CT22 data acquisition unit (Qianpu, Jiangsu, China), and was then processed by an HW2000 chromatography workstation (Qianpu). Separation was carried out in a fused silica capillary (50 μm ID, 365 μm OD, 50.0 cm total length and 40.0 cm effective length, Yongnian Photoconduction Fibre, Hebei, China). Each day before analysis, the capillary was sequentially rinsed, with 1 M NaOH for 30 minutes, triple distilled water for 10 minutes and running buffer for 5 minutes. The running buffer was a mixture of borax (10 mM) and phosphoric acid (20 mM) at pH 8.52. The sample was hydrodynamically injected into the capillary by lifting the sample vial by 15.0 cm for 12 s, and electrophoretic separation was performed at a voltage of 12 kV. For good reproducibility, the capillary tube was rinsed with running buffer solution for 5 minutes between two consecutive runs.

For quantification, a calibration curve was established first by using the standard mixture of GSSG and GSH at varying concentrations (as shown in Figure S9). The upper- and bottom phases were diluted with triple distilled water properly, analyzed with capillary electrophoresis using the same conditions (as shown in Figure S10). The concentrations of GSSG, calculated based on the calibration curve, were 7.6 mM and 137.27 mM, respectively, in upper- and bottom phases.

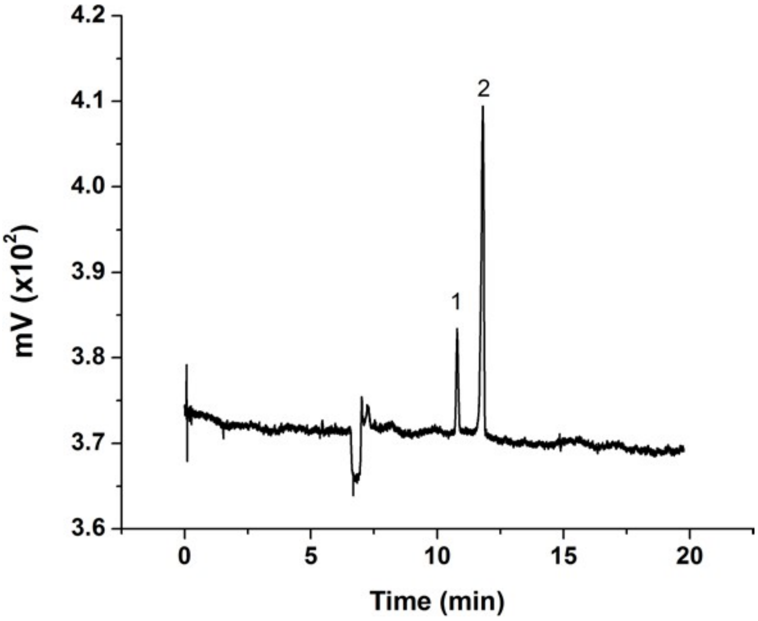

Figure S9 Capillary electrophoresis of the standard mixture of GSH (1) and GSSG (2).

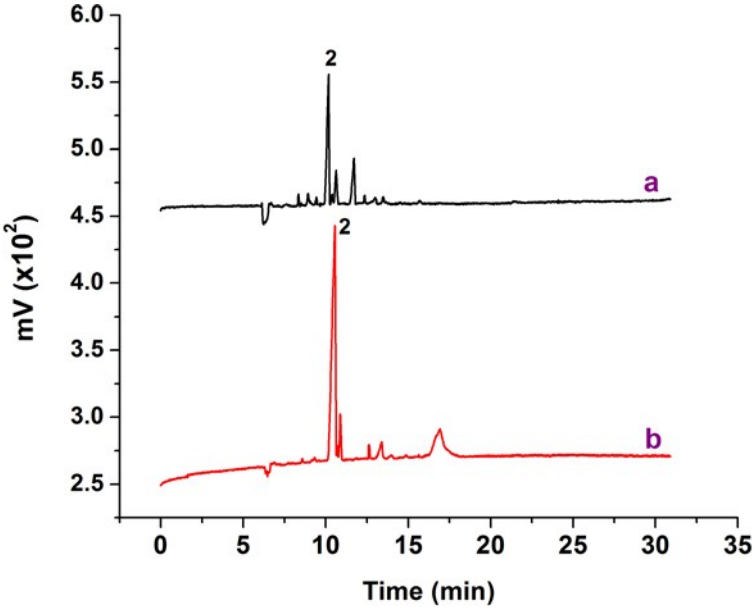

Figure S10 Representative electropherograms of the upper acetonitrile-rich phase (a) and the bottom phase (b). Traces were offset for clarity.

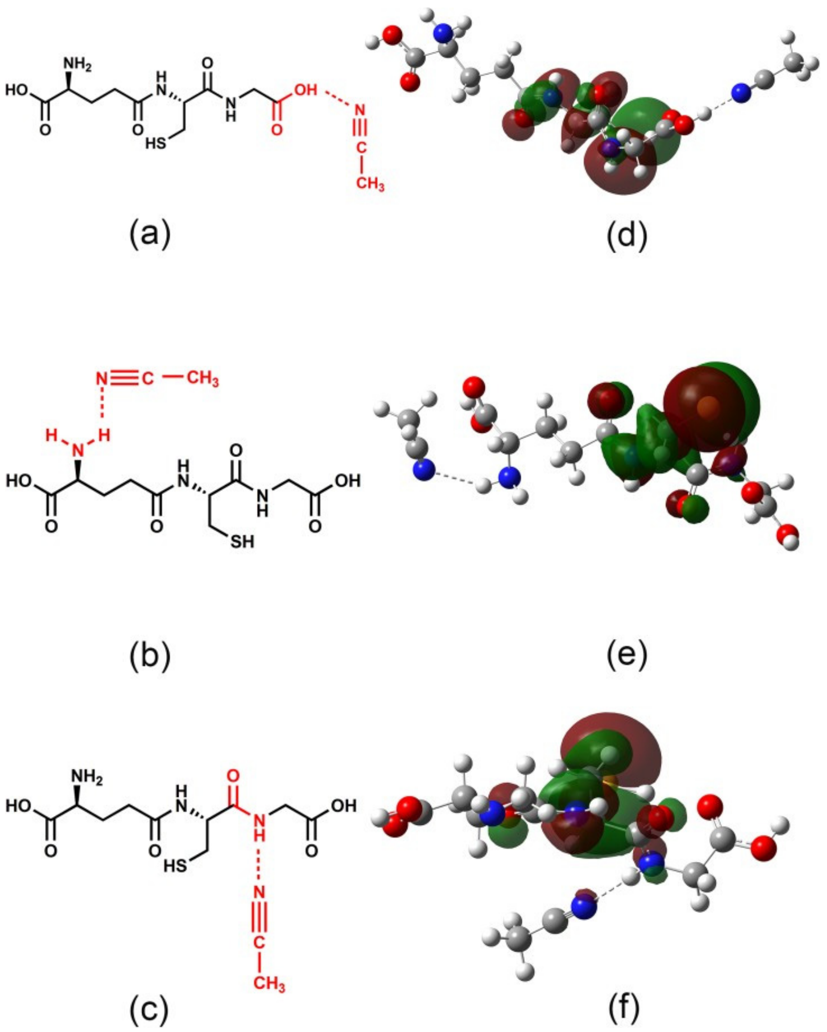

Figure S11 Hypothesized hydrogen bonds formed between acetonitrile and the GSH functional groups: (a) carboxyl group; (b) amine group; (c) amide group. (d)-(f) are the corresponding frontier molecular orbitals of the charge transfers for (a)-(c).
